# Supplementary material for: Patterns of Intron Gain and Loss in Fungi
Source: PLoS Biol. 2004 Nov 30;2(12):e422. doi: 10.1371/journal.pbio.0020422 (PMC532390; doi:10.1371/journal.pbio.0020422)
Supplement: Table S1 — Also available at http://genes.mit.edu/NielsenEtAl/. (4.3 MB ZIP). [file pbio.0020422.st001.zip › NielsenEtAl/html/937.html]

AN6569.1.NCU03836.1.MG06931.1.FG07406.1


```
 CLUSTAL W (1.82) Multiple Sequence Alignments - Introns Inserted


Sequence 1: NCU03836.1	354 aa
Sequence 2: MG06931.1	354 aa
Sequence 3: FG07406.1	363 aa
Sequence 4: AN6569.1	363 aa
Alignment Length: 391 aa
Number Identitical Residues: 243 aa
Alignment Score (without introns) 10358


MG06931.1 	--------------~MAIPSNPAK~RRRIALGCEGSANKLGIGIIAHPPEGEVGDPVVLS
NCU03836.1	--------------~--MP--DAK~GRRIALGCEGSANKLGIGIIAHDP--ITGEALVLS
FG07406.1 	MTPVEFDCLSEPRA1TCICNSPVQ0SSYIALGCEGSANKLGIGVILHTP----TETKILS
AN6569.1  	--------------~---------~--MIAIGLEGSANKLGVGIMLHPK--DGSTPQVLA
          	                            **:* ********:*:: *        . :*:

MG06931.1 	NVRDTFVSPPGTGFLPKDTAAHHRSFFVRVAQQAIRDAGVTVAEVDCICYTKGPGMGAPL
NCU03836.1	NVRDTFVSPPGTGFLPKDTARHHRAYFVRVAKKALALSGVSISEIDCICYTKGPGMGGPL
FG07406.1 	NLRDTFVSPPGTGFLPKDTAAHHRAHFVRLAREALAEAKITPADVDCICYTKGPGMGAPL
AN6569.1  	NIRHTYVSPPGEGFLPKDTARHHRSWVVSLVKKALKEARISVDDVDCICYTKGPGMGAPL
          	*:*.*:***** ******** ***: .* :.::*:  : ::  ::************.**

MG06931.1 	TSTAIGARTLALLWDKPLVGVNHCVGH~IEMGRAITGADNPVVLYVSGGNTQVIAYAEQR
NCU03836.1	TSVAVGARTLALLWGKELVGVNHCVGH~IEMGRAITGASNPVVLYVSGGNTQVIAYAEQR
FG07406.1 	NSVAVAARALSLLWDRPLVGVNHCVGH~IEMGRYITGAENPVVLYVSGGNSQVIAYAEQR
AN6569.1  	QSVAVAARTLSLLWGKELVGVNHCVGH1IEMGRLITGASNPVVLYVSGGNTQVIAYSSQR
          	 *.*:.**:*:***.: ********** ***** ****.***********:*****:.**

MG06931.1 	YRIFGETLDIAVGNCLDRFARTLKISNDPAPGYNIEQLAKQGS-VLLDLPYAVKGMDCSF
NCU03836.1	YRIFGETLDIAVGNCLDRFARTLEISNDPAPGYNIEQLAKQGGRVLLDLPYAVKGMDCSF
FG07406.1 	YRIFGETLDIAVGNCLDRFARTLEISNDPAPGYNIEQLAKKGS-KLLDIPYAVKGMDCSF
AN6569.1  	YRIFGETLDIAVGNCLDRFARTLHISNDPAPGYNIEQLAKKGK-QLVDLPYTVKGMDCSM
          	***********************.****************:*   *:*:**:*******:

MG06931.1 	SGILTRADELAAQMVAKP----------------------DLFTPADLCFTLQETVFAML
NCU03836.1	SGILGRADDLAAQMKAGEPGPD-----------------GEPFTPADLCFSLQETVFAML
FG07406.1 	SGILASADALAAQMKAGA-----------------------DFTPEDLCFSLQETVFAML
AN6569.1  	SGILAAIDALAATYGLNGEQPDEEEDVTDVTPVSDGALESRKPTRADLCFSLQETVFSML
          	****   * ***        ...... :. :. :..: ..   *  ****:******:**

MG06931.1 	VEITERAMAHVGSTQVLIVGGVGSNERLQQMMGAMAKDRGGSVYATDERFCIDNGIMIAH
NCU03836.1	VEITERAMAHVGSNQVLIVGGVGCNERLQEMMGAMAAERGGSVYATDERFCIDNGIMIAH
FG07406.1 	VEITERAMAHVGSSQVLIVGGVGCNERLQEMMGHMARERGGSVYATDERFCIDNGIMIAH
AN6569.1  	VEITERAMAHVGSKEVLIVGGVGCNERLQEMMGIMARDRGGSVHATDERFCIDNGIMIAQ
          	*************.:********.*****:*** ** :*****:***************:

MG06931.1 	AGLLAYETGFRTPLEESTCTQRFRTDEVHVKWRD
NCU03836.1	AGLLAYETGFRTPLDESTCTQRFRTDEVFVKWRD
FG07406.1 	AGLLAYETGFRTSLEESTCTQRFRTDEVFIKWRD
AN6569.1  	AGMLAYKTGFRTPLKESTCTQRFRTDDVFVQWRD
          	**:***:*****.*.***********:*.::***
```
